# Risk Assessment of Heavy Metals, Nitrogen, and Phosphorus in Seawater of Taizhou Bay, China

**DOI:** 10.3390/jox15050143

**Published:** 2025-09-09

**Authors:** Guanghua Xia, Chunling Han, Manting Chen, Guanjie Wang, Kejia Lu, Jianqiang Zhu, Jiachao Yao

**Affiliations:** 1 College of Chemical and Biological Engineering, Zhejiang University, Hangzhou 310030, China; guanghua@zju.edu.cn; 2 Zhejiang Key Laboratory for Restoration of Damaged Coastal Ecosystems, School of Life Sciences, Taizhou University, Taizhou 318000, China; lukejiawork@163.com (K.L.);; 3 Zhejiang Provincial Key Laboratory of Plant Evolutionary Ecology and Conservation, School of Life Sciences, Taizhou University, Taizhou 318000, China; 4 Zhejiang Key Laboratory of Advanced Solid State Energy Storage Technology and Applications, Taizhou Institute of Zhejiang University, Taizhou 318000, China; 5 Ningbo Ningle Construction Engineering Testing Co., Ltd., Ningbo 315042, China; 6 Taizhou Pollution Control Technology Center Co., Ltd., Taizhou University, Taizhou 318000, China; 7 Zhejiang Collaborative Innovation Center for Full-Process Monitoring and Green Governance of Emerging Contaminants, College of Biology and Environmental Engineering, Zhejiang Shuren University, Hangzhou 310015, China

**Keywords:** heavy metal, inorganic pollutant, risk assessment, source, correlation analysis

## Abstract

Heavy metals, nitrogen, and phosphorus play a significant role in the marine ecosystem and human health. In this work, the concentrations of heavy metals, inorganic nitrogen, and phosphorus were determined to assess the distribution characteristics, risk levels, and possible sources in seawater from Taizhou Bay. The concentration ranges of Cu, Pb, Zn, Cd, Hg, As, ammonia, nitrate, nitrite, and phosphate were 1.87–3.65 μg/L, 0.10–0.95 μg/L, 2.98–16.80 μg/L, 0.07–0.38 μg/L, 0.011–0.043 μg/L, 0.93–2.06 μg/L, 0.011–0.608 mg-N/L, 0.012–0.722 mg-N/L, 0.001–0.022 mg-N/L, and 0.004–0.044 mg-P/L, respectively. The ecological risks were evaluated by the single factor index, Nemerow pollution index, and risk quotient. The results indicated that Taizhou Bay is not currently facing ecological risk related to heavy metals, nitrogen, and phosphorus, but the RQ values emphasized the urgency of strengthening continuous monitoring of As, Cu, and Zn. The results of Pearson’s correlation indicated that salinity and chemical oxygen demand had a significant impact on nitrogen and phosphorus but little impact on heavy metals. Principal component analysis was then applied to analyze the probable origins of heavy metals and inorganic pollutants, suggesting that these pollutants were mainly derived from human activities along the bay.

## 1. Introduction

The continuous development of coastal cities and human activities, including reclamation, sewage discharge, fishing, and aquaculture, are constantly expanding into marine areas [1,2]. The environmental problems caused by these activities are becoming increasingly serious, which has affected the stability of ecosystems and human health [3]. Semi-enclosed bays have become the places most closely associated with human activities in marine areas due to favorable geographical conditions [4,5]. They serve as a boundary between land and sea, as well as a buffer zone for filtering pollutants [6]. Thus, it is important to study the distributions and sources of pollutants in semi-enclosed bays, which will help to better assess pollution status, and recognize the relationships between bay development and environmental protection.

Heavy metal pollution in marine environments has become a worldwide concern due to its characteristics of toxicity, non-degradability, and bioaccumulation [7,8]. Heavy metals in seawater can be transferred and accumulated into sediments by adsorption, complexation, and precipitation. In the meantime, heavy metals can be released from sediments into liquid flows to cause seawater contamination as environmental changes (e.g., pH, dissolved oxygen, salinity, and biological activity) [9,10]. Recently, many scholars have focused their research on the distribution and contamination assessment of heavy metals in sediments [11,12]. For instance, Hao et al. [13], investigated the effects of human activities on the ecological risks of heavy metals from sediment in Xincun Bay. The results indicated that mariculture activities could increase the concentrations and corresponding risks of sediment heavy metals. However, compared with the pollutants in sediments, heavy metals dissolved in seawater are much more bioavailable, which are more harmful to marine organisms (e.g., fish and plankton) and will cause greater damage to the marine ecosystem [14,15]. As reported [16,17], cadmium (Cd) has strong bioaccumulation in fish, which can easily lead to calcium loss and cause osteoporosis; lead (Pb) and mercury (Hg) can affect enzyme activity in living organisms even at low levels; excessive copper (Cu) can result in the denaturation of protein structure in marine organisms. Additionally, dissolved inorganic nitrogen and phosphate are also common contaminants in seawater, and often co-exist with heavy metals [18,19]. The relationship between these inorganic pollutants and heavy metals in seawater needs to be further studied [20,21]. Therefore, it is critical to assess the distribution and environmental risk of heavy metals, dissolved inorganic nitrogen, and phosphate in seawater.

Taizhou Bay is a semi-enclosed bay located in Taizhou, China, with a surface area of ~342 km^2^. It is a marine economic development area in Zhejiang Province, a natural spawning and nursery ground for many fishery species, as well as an important transportation port. The continuous development and construction of Taizhou Bay will inevitably aggravate the pollution of the bay. In addition, Taizhou is a typical e-waste disposal site in China. Heavy metals from the dismantling process are likely to enter Taizhou Bay. However, few studies have focused on heavy metal contamination and the potential risk in Taizhou Bay.

In this work, the distribution, main controlling factors, and pollution assessment of six heavy metals (Cu, Pb, Zn, Cd, Hg, and As) and inorganic pollutants (nitrate, nitrite, ammonia, and phosphate) in the seawater of Taizhou Bay were investigated. The main objectives of this study are as follows: (1) to analyze the distributions of heavy metals and inorganic pollutants in the seawater; (2) to evaluate the contamination assessment by the single-factor index and Nemerow pollution index; and (3) to identify the potential sources of contaminants.

## 2. Materials and Methods

### 2.1. Sample Collection and Analysis

**Figure 1:**
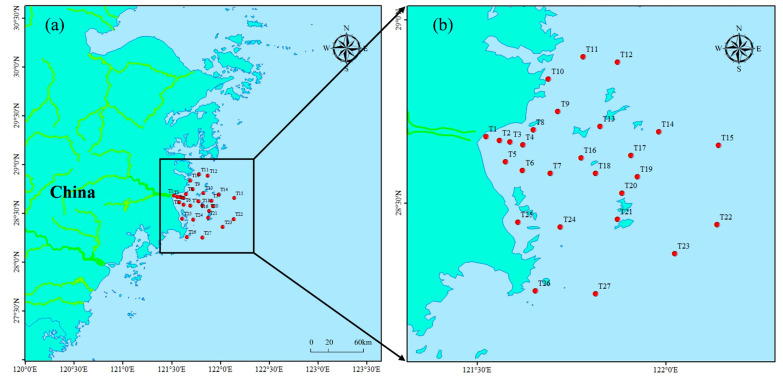
Location of (**a**) Taizhou Bay and (**b**) sampling sites.

Taizhou Bay, a typical semi-enclosed bay located in Taizhou, Zhejiang Province of China, was selected as the study area in this work (Figure 1). The water depth in most areas of Taizhou Bay is ~3 m, and the deepest point reaches ~10 m. This bay connects with the east coast of China. The local society-environment-economy balance of the bay is affected by the daedal interaction of human activities, such as socio-economic development, industrial wastewater discharge, mariculture pollution, and transportation pollutant emissions.

A total of 27 sampling stations were selected in the summer (July 2023) to investigate the pollutant distributions in Taizhou Bay (Figure 1). The 27 sampling points were laid out in a grid pattern, with a denser distribution closer to the coastline and less dense distribution further away from the coastline. The distances from the coastline were relatively larger, with overall distances ranging from 5 to 7 km. In the summer, industrial and agricultural activities around the coast are relatively more active, and the concentration of pollutants also increase accordingly. Therefore, summer was selected to evaluate the pollution situation of the bay. Sample collection, storage, processing, and analysis were strictly in accordance with the Specifications for Oceanographic Survey (GB/T 12763-2007) [22] and the Specification for Marine Monitoring (GB 17378-2007) [23]. Briefly, the distance between stations was approximately 5 to 7 km, while the sampling depth was approximately 10 m. The concentrations of Cu, Pb, Zn, Cd, and As were determined by inductively coupled plasma mass spectrometry (iCAP RQ, Thermo, Waltham, MA, USA). Hg concentration was measured using atomic fluorescence spectrometry (PF52, Puxi, Shanghai, China). The pH value and salinity were analyzed by a portable pH meter and salinity meter, respectively. The basic potassium permanganate method was applied to measure the chemical oxygen demand (COD). The Hg sample needed to be added to sulfuric acid to pH < 2 and stored in a 500 mL glass bottle. The samples of Cu, Pb, Zn, Cd, and As needed to be filtered and added to nitric acid to pH < 2, and stored in a 500 mL polyethylene bottle. The COD sample needed to be added to sulfuric acid until pH < 2, then frozen, and stored in a 500 mL polyethylene bottle. The water sample containing inorganic salts was filtered and frozen for storage in a 500 mL polyethylene bottle. The samples needed to be detected and analyzed within 24 h after sampling. The limits of quantifications (LOQ) were 0.12 μg/L for Cu, 0.07 μg/L for Pb, 0.10 μg/L for Zn, 0.03 μg/L for Cd, 0.05 μg/L for As, and 0.007 μg/L for Hg. The percentages of recoveries were 88.1%~92.4% for Cu, 95.4%~97.2% for Pb, 93.2%~96.3% for Zn, 96.3%~98.2% for Cd, 97.1%~98.8% for As, and 96.4%~98.6% for Hg.

Blank testing and calibration standards were listed as follows: (1) Blank test: at least one blank test should be conducted for each batch of samples during analysis. (2) Quantitative calibration: during continuous injection analysis, for testing of each of the 20 samples, the concentration point in the middle of the calibration curve should be measured once to confirm whether there was a significant change in the calibration curve of the analytical instrument. (3) Precision control: parallel double sample analysis must be performed for each testing item during the analysis of each batch of samples. (4) Accuracy control: when there is a certified standard substance that is the same or similar to the matrix of the tested seawater sample, a certified standard substance sample with a content level equivalent to that of the tested sample should be uniformly inserted for analysis and testing during each batch of sample analysis.

The quality control procedure is summarized as follows: (1) During the sample determination process, blank samples should be added to the testing site. (2) On-site parallel samples: on-site parallel samples should account for 5% to 10% of the total sample size, and at least 2 sets of parallel samples should be taken each time. (3) Equipment material blank: when using new sampling equipment, new containers, and new materials, blank testing of equipment materials should be conducted. (4) The analysis blank should account for 5% of the total number of samples. When the number of samples was less than 20, at least one analysis blank should be included in each batch. (5) Each batch of samples should be tested for the spiked recovery rate based on 2% of the total number of samples (at least 2 samples should be tested if there are less than 10 samples). (6) When the sample size exceeds 20, parallel analysis of 3 samples should be conducted. (7) When the measured value and spiked recovery rate of the quality control sample exceed the control line, the cause should be investigated, and analysis should not be continued until the cause is identified.

The use of blank or certified reference materials was performed as follows: (1) Single-element standard stock solutions have concentrations of 100.0 mg/L for copper, lead, zinc, cadmium, and arsenic, with nitric acid as the solvent. These should be stored at 4 °C for refrigeration, with a validity period of 1 year. (2) The single-element standard stock solution for mercury at 1 ug/L, should be placed in a cool and dry place, avoiding light and high-temperature environments, with a validity period of 1 year.

### 2.2. Heavy Metal Pollution Assessment

The single-factor index (SFI) was conducted to assess the pollution level of contaminations, which is determined as follows [24]:

(1) $$SFI_{i}=C_{i}/C_{n}$$

where *SFI_i_* is the pollution index of contamination *i*. *C_i_* and *C_n_* are the determined pollutant concentration and its corresponding standard value (μg/L), respectively. The standard values for Cu, Pb, Zn, Cd, Hg, As, dissolved inorganic nitrogen (DIN, sum of the concentrations of ammonia, nitrate, and nitrite), and phosphate are 5 μg/L, 1 μg/L, 20 μg/L, 1 μg/L, 0.05 μg/L, 20 μg/L, 0.3 mg-N/L, and 0.03 mg-P/L, respectively, which are obtained from the Sea Water Quality Standard (GB 3097-1997) [25]. According to previous work [26], for the assessment of heavy metals, SFI < 0.2 indicates a normal background level, 0.2 < SFI < 0.6 indicates light pollution, 0.6 < SFI < 1.0 indicates mild pollution, and SFI > 1 indicates strong pollution. As for inorganic pollutants (including nitrogen and phosphate), SFI ≤ 1 indicates compliance with the standard, otherwise it is considered non-compliant.

Then the Nemerow pollution index (P_N_) was applied to assess the status of multiple pollution, and the P_N_ can be calculated as follows [27]:

(2) $$P_{N}=\sqrt{\frac{P_{\max}^{2}+P_{mean}^{2}}{2}}$$

where *P_max_* and *P_mean_* are the maximum and average values of the pollution indexes, respectively.

The risk quotient (RQ) was selected to evaluate the ecological risk level of heavy metals [28]:

(3) $$RQ=C_{i}/PNEC$$

(4) $$PNEC=HC_{5}/SF$$

where *PNEC* is the predicted no-effect concentration (μg/L). *HC*_5_ is the 5th percentile of the species sensitivity distribution model (μg/L), and the values of HC5 for Cu, Pb, Zn, Cd, Hg, and As are 3.46, 234.06, 25.54, 1.07, 1.38, and 0.5, respectively [29]. *SF* represents the safety factor (constant 5) [28].

## 3. Results and Discussion

### 3.1. Characteristics of Seawater Property and Distribution of Heavy Metals

**Figure 2:**
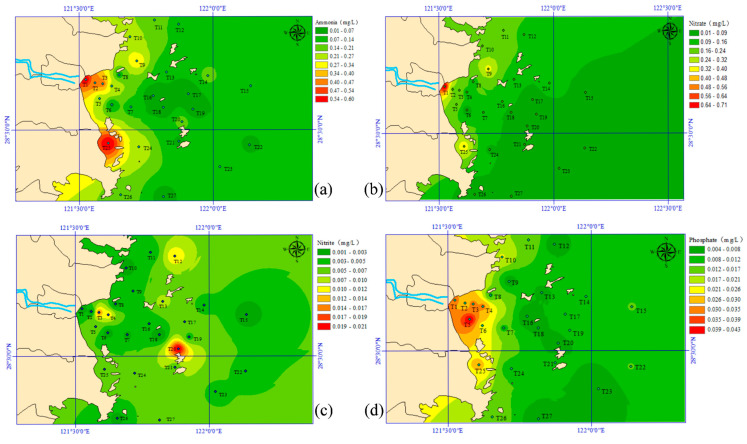
Spatial distribution of (**a**) ammonia, (**b**) nitrate, (**c**) nitrite, and (**d**) phosphate in Taizhou Bay.

**Figure 3:**
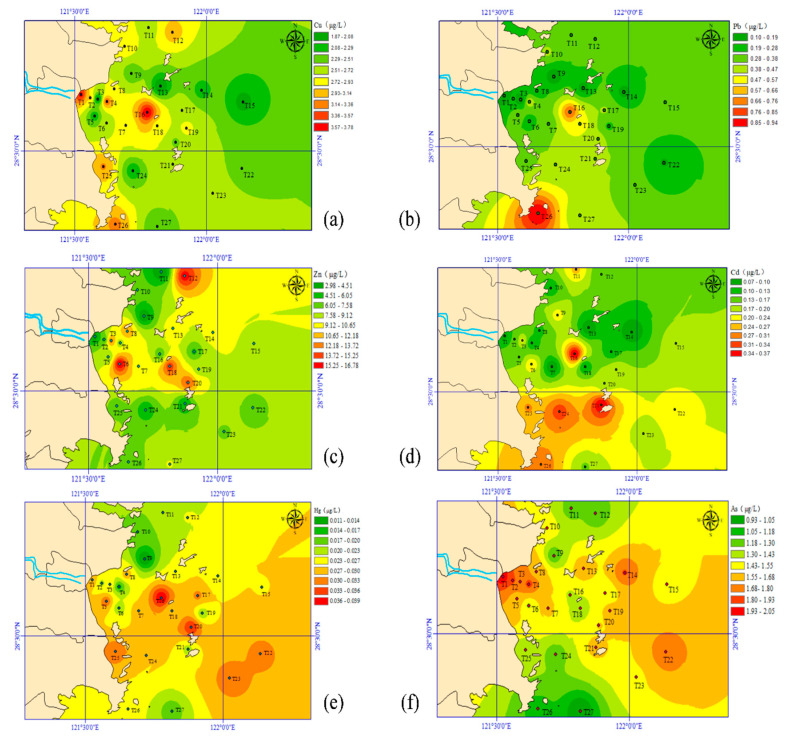
Spatial distribution of (**a**) Cu, (**b**) Pb, (**c**) Zn, (**d**) Cd, (**e**) Hg, and (**f**) As in Taizhou Bay.

Spatial variation of water properties in the surface seawater in Taizhou Bay is displayed in Figure 2. As shown, ammonia, nitrate, nitrite, and phosphate were in the ranges of 0.011–0.608 mg-N/L, 0.012–0.722 mg-N/L, 0.001–0.022 mg-N/L, and 0.004–0.044 mg-P/L, respectively. Their mean concentrations were in the following order: ammonia > nitrate > phosphate > nitrite. Moreover, nitrate and phosphate in the east area (i.e., near station T1) were significantly higher than that of other areas. The high concentration of nitrite was observed in the middle of the bay, near station T20. As for ammonia, station T1 and T25 were determined as the key areas for high concentration. The pollution distribution of the selected heavy metals in Taizhou Bay is shown in Figure 3. The order of the average contents of heavy metals in seawater was Zn > Cu > As > Pb > Cd > Hg. The concentration ranges of Cu, Pb, Zn, Cd, Hg, and As were 1.87–3.65 μg/L, 0.10–0.95 μg/L, 2.98–16.80 μg/L, 0.07–0.38 μg/L, 0.011–0.043 μg/L, and 0.93–2.06 μg/L, respectively. The distribution characteristics of Hg and As were similar, with high concentrations in the east and west of the Taizhou Bay. As for Pb and Cd, they are mainly distributed in the middle and southwest. However, the distribution of Zn and Cu were irregular, i.e., a patchy distribution of high-value regions emerged. A high Zn content was found in the west and north, while the high concentration of Cu was mainly concentrated near the land. Additionally, Table 1 summarizes some reference results of heavy metals in the seawater of other regions. The Cu, Pb, Zn, Cd, and As concentrations in Taizhou Bay were similar to those in Hangzhou Bay but lower than those of Bohai Bay. The Hg concentration was comparable with other regions in China. The Zn and Cd levels were also comparable with the Yellow Sea, but Cu, Pb, and As concentrations were much lower than that of the Yellow Sea.

### 3.2. Risk Assessment of Heavy Metals

The SFI values for six heavy metals were calculated to evaluate the pollution level in seawater. As shown in Table 2, the average SFI values of Cd and As were calculated as 0.19 and 0.08, respectively, indicating that the seawater was unpolluted. However, light pollution levels emerged, which were exhibited by the average SFI values of Cu (0.54), Pb (0.34), Zn (0.43), and Hg (0.53). In addition, the SFI values of some stations had exceeded 0.6, reaching a mild pollution level, e.g., T12, T16, and T25. Furthermore, the SFI values of 1.15 and 0.53 were obtained for DIN and phosphate, respectively. The results indicated that the phosphate concentration met the seawater quality standard, while the DIN did not meet it. This might be related to the use of nitrogen-containing chemical reagents in enterprises (e.g., pharmaceutical and chemical enterprises, mechanical processing enterprises, and electroplating enterprises) and the application of nitrogen fertilizers in farmland along the bar, which would discharge DIN. Additionally, the Nemerow pollution index (P_N_) was selected to assess the multiple effects of heavy metals and inorganic pollutants on water quality. The P_N_ risk levels can be classified into five grades: P_N_ ≤ 0.7 represents safe; 0.7 < P_N_ ≤ 1 represents warming threshold; 1 < P_N_ ≤ 2 represents slight contamination; 2 < P_N_ ≤ 3 represents moderate contamination; and P_N_ > 3 represents severe contamination [35]. As shown in Table 2, the P_N_ values of Cu (0.66), Zn (0.67), Cd (0.30), As (0.09), and phosphate (0.03) were all below 0.7, indicating that the ecological risk was low. In terms of Pb, Hg, and DIN, the P_N_ values were calculated as 0.71, 0.72, and 0.96 respectively, suggesting a warming threshold level. Considering the acceptable levels of pollution exposure for aquatic organisms, the risk quotient (RQ) was used to assess Taizhou Bay [36]. The RQ can be classified into three levels, i.e., light risk (RQ ≤ 0.1), mild risk (0.1 < RQ ≤ 1), and strong risk (RQ > 1). Table 2 shows that the order of the RQ value was As (15.11) > Cu (3.88) > Zn (1.67) > Cd (0.89) > Hg (0.10) > Pb (0.01). The results of the RQ values emphasized the urgency of strengthening continuous monitoring of As, Cu, and Zn in Taizhou Bay. Excessive levels of these heavy metals might inhibit the photosynthesis of phytoplankton, and suppress the metabolic functions of marine organisms, thereby affecting the structure of the marine ecosystem. In addition, raising concerns about the potential ecological risk of Cd is required.

### 3.3. Correlation Between Heavy Metals and Inorganic Pollutants

The Pearson’s correlation coefficients between the concentrations of heavy metals and inorganic pollutants are listed in Table 3. As shown, a significant correlation was observed among nitrate, ammonia, and phosphate, suggesting that these three pollutants may have the same source. As for heavy metals, Pb showed high positive correlations with Cu and Cd, while showing a negative correlation with As. In addition, weak correlations between Hg and Cu, Pb, Zn, Cd, and As emerged, i.e., Hg might have a different source than other heavy metals. Generally, Pb, Cu, and Cd were mainly related to the electroplating industry, surface treatment industry, and mechanical processing industry in coastal industries, while Hg mainly came from coal-fired power plants. A similar phenomenon was also found for Zn. Compared with other regions in China, no significant correlation among the heavy metals was observed in this work. However, compared with other bays in Zhejiang Province [34], similar results were presented. This phenomenon might be related to the diversity and complexity of enterprises near the coast of Taizhou Bay, resulting in different sources of heavy metals. Turning to the relationship between heavy metals and inorganic pollutants, there was significant correlation between phosphate and As with a correlation coefficient of 0.450. However, ammonia, nitrate, nitrite, and phosphate showed weak correlations with other heavy metals, showing that inorganic pollutants were not the chief factors that affect the distribution of heavy metals in Taizhou Bay. Additionally, the relationships among water properties, heavy metals, and inorganic pollutants were described. The results suggested that nitrate, ammonia, phosphate, and As concentrations were significantly negatively related to salinity, while a positive relationship emerged for Pb. Meanwhile, the COD reflected a positive effect on nitrate, ammonia, and As.

### 3.4. Source Identification of Contaminants in Seawater

**Figure 4:**
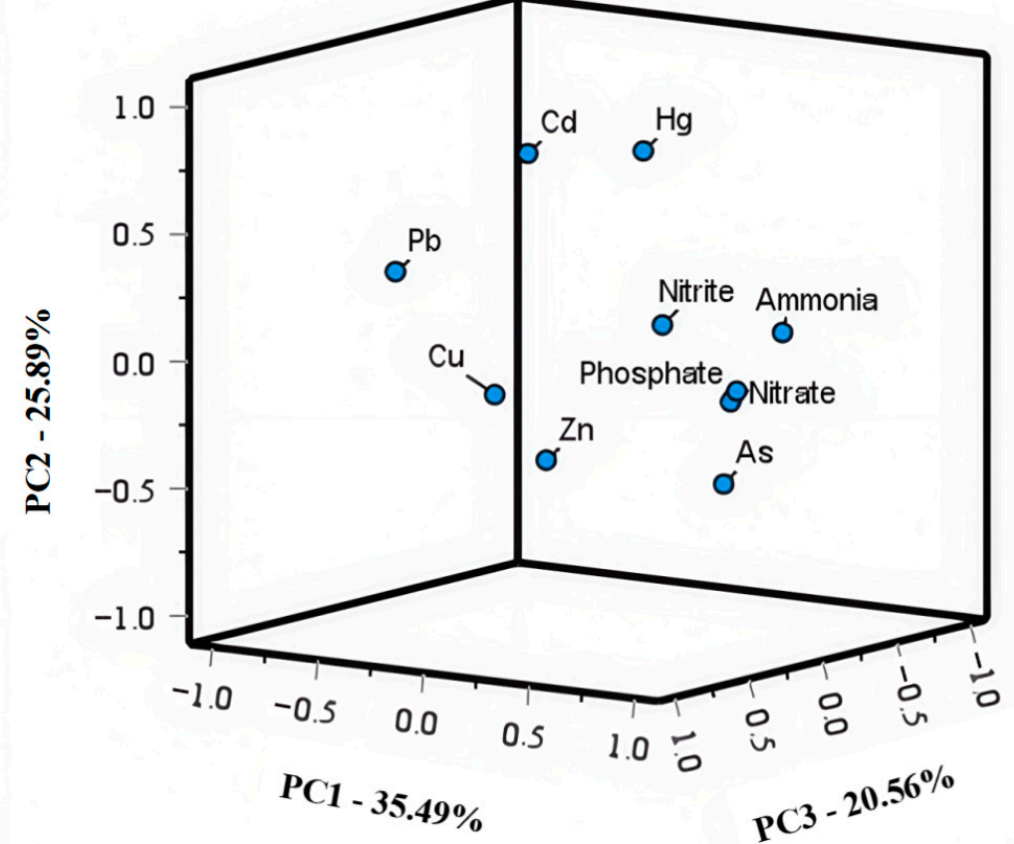
Principal component loading diagram for concentrations of heavy metals and inorganic pollutants.

**Figure 5:**
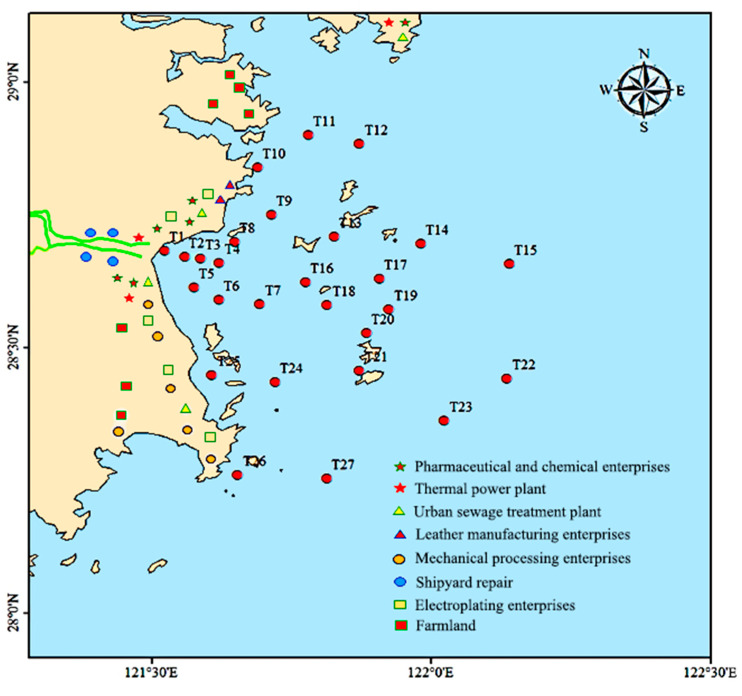
Human activities along Taizhou Bay.

Subsequently, principal component analysis (PCA) was applied to analyze the probable origins of heavy metals and inorganic pollutants [37]. The results of PCA for heavy metals, nitrogen, and phosphate are displayed in Table 4 and Figure 4. As shown, three principal components with high eigenvalues (i.e., >1) were extracted, and 81.94% of total variance emerged. PC1 contributed to 35.49% of the total variance, and high positive loadings of 0.84, 0.81, and 0.72 were obtained for nitrate, ammonia, and phosphate, respectively. Combined with the distribution characteristics of these pollutants near the land (Figure 2), it indicated that these pollutants were mainly from anthropogenic activities, e.g., coastal farmland, and pharmaceutical and chemical enterprises [38]. In addition, the contributions of PC2 (25.89%) contained Cu, Pb, and Cd, which exhibited high loadings. These heavy metals were highly related with electroplating enterprises, ship activity, and industrial and city sewage [39]. Meanwhile, strong significant correlations were also observed among Cu, Pb, and Cd (Table 3), suggesting that PC2 comprised land-sourced pollutant emission and shipping waste. As for PC3, Hg showed a high loading of 0.739. Except for atmospheric deposition, Hg could be mainly contributed to by aquaculture, thermal power plants, and electroplating enterprises [40]. Due to China being a populous country, a large number of people are concentrated in the area near the bay, i.e., there are numerous human activities near the bay. To better trace the source of the pollutants, human activities along Taizhou Bay are displayed in Figure 5. As shown, pharmaceutical and chemical enterprises, thermal power plants, urban sewage treatment plants, leather manufacturing enterprises, mechanical processing enterprises, shipyard repair, electroplating enterprises, and farmland were located along the bay. These activities were closely related to heavy metals, nitrogen, and phosphate.

## 4. Conclusions

In this study, the distribution of heavy metals, nitrogen, and phosphorus, and their pollution risks in the seawater of Taizhou Bay were investigated. The order of the average contents of heavy metals in seawater was Zn > Cu > As > Pb > Cd > Hg, while the order of ammonia > nitrate > phosphate > nitrite was shown for inorganic pollutants. The results of ecological risk assessment indicated that the pollution level of heavy metals and inorganic pollutants in Taizhou Bay was low. In addition, the results of Pearson’s correlation indicated that nitrogen and phosphorus were correlated to salinity and chemical oxygen demand. Three principal components were then identified, and human activities were determined as the main factor that contributed to heavy metals and inorganic pollutants. These findings emphasized the importance of further controlling heavy metals and inorganic pollutants in Taizhou Bay, which would help decision makers to formulate related regional reduction targets and control methods. In addition, it is necessary to strengthen monitoring of the pollution discharge of enterprises around the bay in the future.

## Figures and Tables

**Table 1:** Comparison of heavy metals in seawater of other regions in China (μg/L).

Region	Cu	Pb	Zn	Cd	Hg	As	Ref.
Zhanjiang Bay, China	4.00	0.25	16.74	0.08	/	/	[30]
Bohai Bay, China	0.16–7.17	0.17–9.55	17.3–90	0.02–0.68	0.003–0.36	0.25–4.02	[31]
Hangzhou Bay, China	2.13–4.59	0.21–0.48	7.81–20.34	0.05–0.28	0.03–0.09	1.08–2.57	[32]
Yellow sea, China	74.56	1.65	13.47	0.2	/	33.80	[33]
East sea, China	0.10–6.40	0.16–3.60	2.50–24.0	/	0.001–0.11	0.85–4.20	[34]
Taizhou Bay, China	1.87–3.65	0.10–0.95	2.98–16.80	0.07–0.38	0.011–0.043	0.93–2.06	This work

**Table 2:** The risk assessment of heavy metals, DIN, and phosphate in seawater by SFI, P_N_, and RQ.

Heavy Metals	Cu	Pb	Zn	Cd	Hg	As	DIN	Phosphate
SFI	0.54	0.34	0.43	0.19	0.53	0.08	1.15	0.53
P_N_	0.66	0.71	0.67	0.30	0.72	0.09	0.96	0.03
RQ	3.88	0.01	1.67	0.89	0.10	15.11	/	/

**Table 3:** Correlation analysis of the heavy metals and physicochemical parameters.

	pH	Salinity	COD	Nitrate	Nitrite	Ammonia	Phosphate	Cu	Pb	Zn	Cd	Hg	As
pH	1												
Salinity	0.125	1											
COD	−0.134	−0.713 **	1										
Nitrate	−0.119	−0.519 **	0.471 *	1									
Nitrite	−0.186	0.039	−0.123	−0.147	1								
Ammonia	−0.394 *	−0.463 *	0.483 *	0.768 **	0.141	1							
Phosphate	0.015	−0.382 *	0.655 **	0.450 *	−0.032	0.616 **	1						
Cu	−0.099	−0.143	0.11	0.349	−0.254	0.166	0.096	1					
Pb	0.058	0.533 **	−0.338	−0.367	−0.072	−0.32	−0.169	0.462 *	1				
Zn	0.365	0.021	−0.07	−0.366	0.324	−0.298	−0.117	−0.173	−0.057	1			
Cd	−0.282	0.295	0.036	−0.199	0.04	−0.033	−0.129	0.091	0.409 *	−0.295	1		
Hg	−0.177	0.111	−0.066	−0.085	0.036	0.095	−0.108	−0.207	0.061	−0.19	0.318	1	
As	0.012	−0.693 **	0.429 *	0.329	0.042	0.318	0.450 *	0.067	−0.471 *	−0.043	−0.381 *	−0.21	1

** Correlation is significant at the 0.01 level (two-tailed). * Correlation is significant at the 0.05 level (two-tailed).

**Table 4:** Principal component analysis of heavy metals and inorganic pollutants.

Parameter	PC1	PC2	PC3
Nitrate	0.842	0.281	−0.001
Nitrite	−0.061	−0.411	0.397
Ammonia	0.813	0.232	0.323
Phosphate	0.719	0.079	0.030
Cu	0.180	0.612	−0.592
Pb	−0.555	0.570	−0.292
Zn	−0.275	−0.674	−0.216
Cd	−0.383	0.602	0.382
Hg	−0.183	0.268	0.739
As	0.679	−0.304	−0.135
Eigenvalue	3.549	2.589	2.056
% of total variance	35.491	25.893	20.560
Cumulative % of variance	35.491	61.384	81.940

## Data Availability

The original data presented in this study are included in the article. Further inquiries can be directed to the corresponding authors.
